# The lung microbiome in end-stage Lymphangioleiomyomatosis

**DOI:** 10.1186/s12931-021-01873-y

**Published:** 2021-10-26

**Authors:** Julie Ng, Gustavo Pacheco-Rodriguez, Lesa Begley, Yvonne J. Huang, Sergio Poli, Mark A. Perrella, Ivan O. Rosas, Joel Moss, Souheil El-Chemaly

**Affiliations:** 1 Department of Medicine, Division of Pulmonary and Critical Care Medicine, Brigham and Women’s Hospital, Harvard Medical School, 75 Francis Street, Boston, MA 02115 USA; 2 Pulmonary Branch, National Heart, Lung, and Blood Institute, National Institutes of Health, Bethesda, MD USA; 3 Department of Medicine, Division of Pulmonary and Critical Care Medicine, University of Michigan, Ann Arbor, MI USA; 4 Department of Internal Medicine, Mount Sinai Medical Center, Miami, FL USA; 5 Department of Medicine, Section of Pulmonary and Critical Care Medicine, Baylor College of Medicine, Houston, TX USA

**Keywords:** Lymphangioleiomyomatosis, Microbiome

## Abstract

Lymphangioleiomyomatosis (LAM) is a progressive cystic lung disease with mortality driven primarily by respiratory failure. Patients with LAM frequently have respiratory infections, suggestive of a dysregulated microbiome. Here we demonstrate that end-stage LAM patients have a distinct microbiome signature compared to patients with end-stage chronic obstructive pulmonary disease.

## Main text

Lymphangioleiomyomatosis (LAM) is a progressive cystic lung disease primarily affecting young women. It can occur sporadically or in association with tuberous sclerosis complex (TSC), and is characterized by cells with mutations in the *TSC1* or *TSC2* genes leading to hyperactivation of mechanistic target of rapamycin (mTOR) signaling [1]. Data from the National Heart, Lung, and Blood Institute (NHLBI) National LAM Registry demonstrate that menopausal status and baseline pulmonary disease severity were the most predictive risk factors for progression to death or transplantation [2]. While mortality in LAM patients is clearly driven primarily by respiratory failure, there remains limited phenotypic or biological markers that correlate with pulmonary function decline or disease progression.

Patients with LAM have a high burden of respiratory infections. In a meta-analysis of 11 studies, the incidence rate of respiratory infections was 43.4 and 58.8 per 100 patients-years in LAM patients receiving mTOR inhibitors or placebo, respectively, with an incidence-rate ratio for respiratory infection of 0.71 (95% CI 0.50–1.02), suggesting that the use of mTOR inhibitors does not increase the risk of respiratory infections and may even be protective [3]. Notably, this is comparable to patients with COPD treated with placebo in studies of the phosphodiesterase inhibitor roflumilast, which demonstrated a 56.9 per 100 patient-years incidence rate of COPD exacerbations [4].

The frequent respiratory infections irrespective of treatment in LAM patients suggests that a dysregulated microbiome could be contributing to disease heterogeneity [3]. For example, in patients with COPD, exacerbation subtypes demonstrate distinct bacterial compositions [5]. Additionally, dysregulation of pulmonary microbiota in murine models of chronic inflammatory lung disease indicate that host-microbial cross-talk can promote inflammation and contribute to disease pathogenesis [6]. The pulmonary microbiome represents a promising, untapped modifiable target for identifying patients at risk for respiratory function decline. To investigate this, we sought to identify potential microbial community structures that could be associated with patients with LAM or COPD at high risk for disease progression or death, which was defined as those whose lung disease have progressed sufficiently to require lung transplantation. This study was approved by the Mass General Brigham Institutional Review Board (protocol 2015P001390) and NHLBI Institutional Review Board (protocols 95-H-0186, 96-H-0100). Samples at the National Institutes of Health (NIH) included de-identified specimens from National Disease Research Interchange (NDRI), or patients who were previously enrolled in NIH protocols. The extramural investigators were not provided with the identity of patients seen at the NIH. Twenty-five tissue specimens (N = 15 [LAM], N = 10 [COPD]) from lung explants from all female patients were obtained at the time of transplantation. All samples were frozen prior to DNA extraction. Twenty samples (N = 11 [LAM], N = 9 [COPD]) were sent to the University of Michigan for microbial analysis for DNA extraction and analyses as previously described [7–9]. DNA was pre-extracted from 5 samples as a pilot. DNA from all 25 tissue samples and 3 extraction controls underwent 16S rRNA gene sequencing of the V4 region on the Illumina MiSeq platform. Sequencing data were deposited in the National Center for Biotechnology Information Sequence Read Archive (BioProject: PRJNA706726). Only samples with > 1000 reads were utilized (N = 12 [LAM], N = 9 [COPD]) for further analyses. Bacterial sequences were classified into operational taxonomic units (OTUs), identified based on the SILVA reference database. Data analyses were performed on all samples with > 1000 reads with and without the pre-extracted DNA samples, demonstrating similar trends. We proceeded with reporting the results of all samples (N = 21) with > 1000 reads regardless of extraction method. The number of sequence reads did not differ significantly between the two groups.

**Fig. 1:**
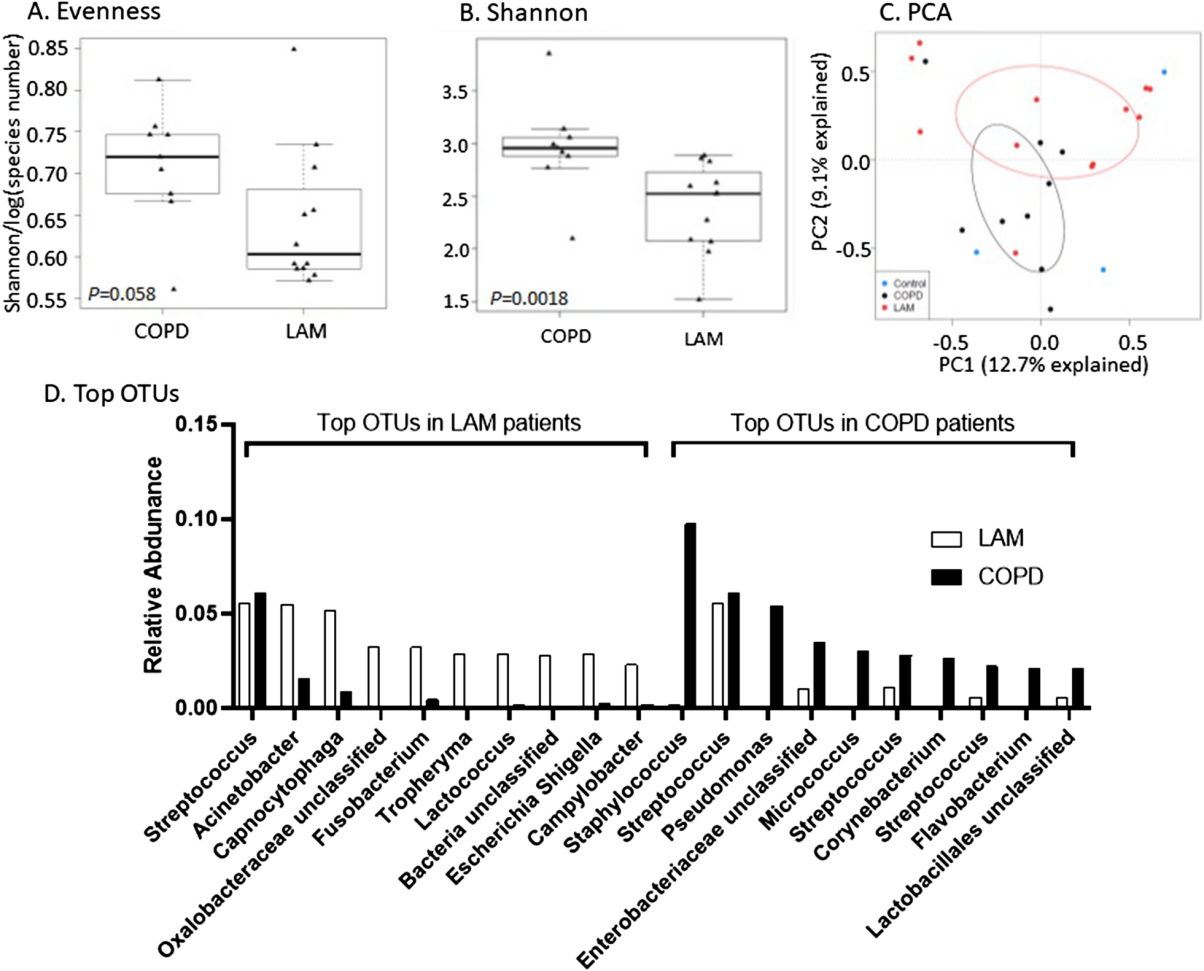
Microbiome in lung explants of patients with LAM compared to COPD. **A** Comparison of Pielou’s evenness index between lung explant specimens with > 1000 reads from COPD (n = 9) and LAM (n = 12) patients. Evaluation of alpha diversity in lung tissue samples from patients with LAM and COPD by (**B**) Shannon Index. Data were assessed by Wilcoxon rank sum and significant comparisons denoted by ** (*P* = 0.0018). **C** Beta-diversity analyses were performed based on Hellinger-transformation of the relative abundance data and visualized in a principal component analysis (PCA) plot. The second principal component (PC2) explained 9.1% of the variation in lung bacterial composition and distinguished microbiota differences between COPD (black circles) and LAM (red circles) samples. Extraction controls (blue circles) did not cluster independently. Data were analyzed by PERMANOVA test (*P* = 0.009). Bacterial sequences were classified into operational taxonomic units (OTU) and OTUs comprising > 0.1% of all individual communities in the dataset were utilized for analyses. **D** The top 10 represented bacteria by average rank relative abundance between LAM (white bars) and COPD (black bars) lung tissue specimens are presented

Evaluation of the microbiome from end-stage LAM versus COPD lungs revealed distinct microbial community structures between the two disease states. Evenness, or the relative amounts of each species present in an environment, was calculated using the Pielou’s evenness index (Fig. 1A), demonstrating a trend towards lower evenness in LAM patients (*P* = 0.058). There was no difference in the number of distinct species among disease states (data not shown). Utilizing alpha-diversity measures that account for both the abundance and richness of the microbial community within a given environment, we found that patients with end-stage LAM had significantly lower alpha diversity (Fig. 1B) by Shannon (*P* = 0.0018), Simpson (*P* = 0.018) and Inverse Simpson (*P* = 0.018) indices, suggesting that patients with end-stage LAM have lower microbial species diversity than patients with end-stage COPD.

Principal component analysis of the taxonomic composition of the lung tissue microbiomes revealed distinct clustering and separation between LAM and COPD patients (Fig. 1C) along the second principal component (PC2) axis, which explained 9.1% of the variation in lung bacterial composition. Extraction controls did not cluster independently with spurious OTUs identified, suggesting the absence of systematic contamination. *Proteobacteria* predominated the lung microbiota of both end-stage LAM and COPD patients. This is consistent with previously published reports of proportional increases in *Proteobacteria* with COPD exacerbation events and Global Initiative for COPD (GOLD) stage, likely reflecting disease severity [10, 11]. However, the top ten represented bacteria by average rank abundance shared only one common organism between the two patient populations—*Streptococcus* (Fig. 1D). *Staphylococcus* and *Pseudomonas* were relatively more abundant in end-stage COPD lungs, whereas *Acinetobacter* predominated in LAM patients.

This is the first study to examine the lung microbiome of end-stage LAM patients as a potential feature of severe disease, which was marked by decreased microbial diversity, and possibly a distinct microbial signature in LAM compared to COPD patients. Both end-stage diseases demonstrated microbiota that were distinct from that found in non-smoker control lungs, which have been associated with *Comamonadaceae, Brevundimonas* and *Diaphorobacter* species [11], with both young and elderly sputum samples from healthy individuals consisting of mainly microbes from the *Firmicutes* phylum [12]. Alterations in the microbiome can result from three major perturbations: (1) the introduction of microbes through inhalation or aspiration, (2) changes in the elimination of microbes through cough, mucociliary clearance, or host immune response, or (3) local environmental growth conditions [13, 14]. In patients with COPD, a shift towards *Proteobacteria* phylum was observed in patients during exacerbations [15] and after rhinovirus infection [16], which in turn has been associated with high mortality and accelerated pulmonary function decline [17]. The findings from our cohort of end-stage COPD patients support this finding, and extends it to patients with LAM, suggesting that frequent infections in LAM [3] may contribute to previously unrecognized perturbations in the lung microbiome. In line with previously published studies, the microbiome of our end-stage COPD patients were enriched for *Pseudomonas* [18], *Streptococcus* [5] and *Staphylococcus* [19]—common pathogenic organisms. In contrast, patients with end-stage LAM had a microbiome that was enriched for *Acinetobacter*, which has also been observed in the allograft microbiome of single lung transplant recipients [20]. This suggests that in addition to infections, immunomodulation may also play a role in shaping the microbiome of patients with LAM. Clarifying the relative contributions of infections and immunomodulation are important future research directions. This can be done by longitudinally evaluating the microbiome in bronchoalveolar lavage samples of different stages of disease, with or without mTOR inhibitor use, in LAM patients. Additionally, enriching for *Acinetobacter* in murine models of LAM [21] and studying its effect on airspace enlargement in vivo, or investigating the impact of inactivated *Acinetobacter* [22] on the growth of TSC2-deficient cells [23] in vitro may represent complimentary mechanistic studies to further delineate the potential impact of the microbiome on patients with LAM.

This analysis had several limitations: the small sample size due to the rarity of the LAM patient population, the absence of clinical data, the heterogeneity of lung disease in patients with COPD, and the lack of longitudinal samples limited our ability to determine if the observed dysregulated microbiome was a consequence of immunomodulator therapy, repeated infections, antibiotic use, or a specific feature of severe progressive LAM. The use of end-stage tissues also may not necessarily represent active pathology, although diffuse disease involvement throughout the lungs may minimize sampling bias in small potentially heterogenous tissue samples. Our study was strengthened by using lung tissue as opposed to bronchoalveolar lavage or sputum samples, which can be contaminated by upper respiratory tract or oral flora, although extraction of adequate amounts of microbial DNA can be challenging in samples dominated by host DNA. The results of this exploratory study suggest that further evaluation of the contribution of microbial populations in LAM patients may represent a novel area of research for the field.

## Data Availability

The datasets generated and/or analyzed during the current study will be available in the National Center for Biotechnology Information Sequence Read Archive (BioProject: PRJNA706726) on publication of this manuscript.

## Abbreviations

COPD: Chronic obstructive pulmonary disease
GOLD: Global Initiative for COPD
LAM: Lymphangioleiomyomatosis
mTOR: Mechanistic target of rapamycin
NDRI: National Disease Research Interchange
NHLBI: National Heart, Lung, and Blood Institute
NIH: National Institutes of Health
OUT: Operational taxonomic units
PC: Principal component
TSC: Tuberous sclerosis complex
